# Vildagliptin, but not glibenclamide, increases circulating endothelial progenitor cell number: a 12-month randomized controlled trial in patients with type 2 diabetes

**DOI:** 10.1186/s12933-017-0503-0

**Published:** 2017-02-23

**Authors:** Alessandra Dei Cas, Valentina Spigoni, Monia Cito, Raffaella Aldigeri, Valentina Ridolfi, Elisabetta Marchesi, Michela Marina, Eleonora Derlindati, Rosalia Aloe, Riccardo C. Bonadonna, Ivana Zavaroni

**Affiliations:** 1 Endocrinology and Metabolic Diseases, Department of Medicine and Surgery, University of Parma, Via Gramsci, 14, 43126 Parma, Italy; 2 Azienda Ospedaliero-Universitaria of Parma, Parma, Italy; 3 Biochemistry, Azienda Ospedaliero-Universitaria of Parma, Parma, Italy

**Keywords:** EPC, SDF-1α, DPP-4 inhibitors, Cardiovascular risk

## Abstract

**Background:**

Fewer circulating endothelial progenitor cells (EPCs) and increased plasma (C-term) stromal cell-derived factor 1α (SDF-1α), a substrate of DPP-4, are biomarkers, and perhaps mediators, of cardiovascular risk and mortality. Short-term/acute treatment with DPP-4 inhibitors improve EPC bioavailability; however, long-term effects of DPP-4i on EPCs bioavailability/plasma (C-term) SDF-1α are unknown.

**Methods:**

Randomized (2:1) open-label trial to compare the effects of vildagliptin (V) (100 mg/day) vs glibenclamide (G) (2.5 mg bid to a maximal dose of 5 mg bid) on circulating EPC levels at 4 and 12 months of treatment in 64 patients with type 2 diabetes in metformin failure. At baseline, and after 4 and 12 months, main clinical/biohumoral parameters, inflammatory biomarkers, concomitant therapies, EPC number (CD34^+^/CD133^+^/KDR^+^/10^6^ cytometric events) and plasma (C-term) SDF-1α (R&D system) were assessed.

**Results:**

Baseline characteristics were comparable in the two groups. V and G similarly and significantly (p < 0.0001) improved glucose control. At 12 months, V significantly increased EPC number (p < 0.05) and significantly reduced (C-term) SDF-1α plasma levels (p < 0.01) compared to G, with no differences in inflammatory biomarkers.

**Conclusions:**

V exerts a long-term favorable effect on EPC and (C-term) SDF-1α levels at glucose equipoise, thereby implying a putative beneficial effect on vascular integrity.

*Trial registration* Clinical Trials number: NCT01822548; name: Effect of Vildagliptin vs. Glibenclamide on Circulating Endothelial Progenitor Cell Number Type 2 Diabetes. Registered 28 March, 2013

## Background

Endothelial progenitor cells (EPCs) are key players in the process of endothelial repair/replacement as they substantially contribute to endothelial homeostasis and neoangiogenesis in response to different detrimental cues [1]. In type 2 diabetes, EPC number is reduced, presumably resulting in an unbalance between the processes of endothelial injury and repair [2]. Fewer EPCs are a biomarker, and perhaps a pathogenetic factor, of vascular damage also in diabetes and the presence of diabetic macrovascular complications is associated with a further reduction in circulating EPC number [3]. Importantly, lower EPC number has been also shown to predict incident cardiovascular (CV) disease [4] and death [5]. Based on the previous considerations, anti-diabetic drugs effective in enhancing EPC number may convey an added value, beyond glucose control, to reduce CV risk and to prevent CV disease in patients with type 2 diabetes. A number of anti-diabetic drugs, namely pioglitazone [6] and insulin [7], and statins [8] have been shown to improve EPC biology in ex vivo studies. Recent evidence points to a potential role of dipeptidyl peptidase-4 inhibitors (DPP-4i) in increasing circulating EPC number. Short-term treatment (4 weeks) with sitagliptin in a small non-randomised trial [9] and acute treatment (4 days) with linagliptin improved EPC bioavailability [10]. A subsequent study investigated the mid-term effects of sitagliptin in boosting EPC levels, but the reported results may be potentially attributable to a regression to the mean effect [11]. Long-term effects of DPP-4i—and specifically of the DPP-4i vildagliptin—in enhancing EPCs bioavailability are unknown.

Importantly, DPP-4 is a ubiquitously expressed protein which cleaves _NH2_-terminal dipeptides from various proteins. One relevant DPP-4 substrate, besides glucagon-like peptide-1 (GLP-1), is stromal cell-derived factor 1α (SDF-1α). SDF-1∝ may act as a double edge sword with respect to the CV system [12]. On the one hand, it is a potent chemokine stimulating stem cell mobilization, including EPC, from the bone marrow and, it plays a positive role in the angiogenic process following acute ischemic injury [13]. On the other hand, C-terminal (C-term) SDF-1α has recently emerged as a robust biomarker of CV diseases and mortality. In the Framingham Heart Study, higher (C-term) SDF-1α levels were associated with the presence of several CV risk factors and independently with heart failure and with 10 year all-cause mortality [14]. In the chronic renal insufficiency cohort higher (C-term) SDF-1∝ levels predicted increased 6-year mortality [15].

The present investigation, therefore, was undertaken to compare long-term effects of the DPP-4i (vildagliptin) vs sulfonylurea (glibenclamide) (to achieve glycaemic equipoise) in patients with type 2 diabetes on a. the bioavailability of EPCs as key regulators of endothelial repair processes b. plasma levels of (C-term) SDF-1α as a biomarker of CV risk and mortality.

## Methods

We conducted a randomized (2:1), open-label active-treatment-controlled clinical trial (NCT01822548) to compare the effects of vildagliptin (V) (100 mg/daily) vs glibenclamide (G) (2.5 mg bid to a maximal dose of 5 mg bid) on circulating EPC and (C-term) SDF-1∝ levels at 4 and 12 months of treatment.

### Subjects

Individuals with type 2 diabetes were recruited in the outpatient Diabetes Unit of Parma University Hospital among those participating in a study funded by the Italian Research program Regione-Università 2007–2009. Eligible subjects were men and women aged ≥35 years, with an established diagnosis of type 2 diabetes (ADA criteria) [16], with at least 1 year of disease duration at the time of the screening visit, in metformin failure [HBA1C 7–9%; 53–75 mmol/mol] and BMI ≥ 20 or ≤40 kg/m^2^. Other inclusion criteria were: treatment with metformin in monotherapy at a stable dose of at least 1.5 g/day (or maximum tolerated dose) in the 3 months prior to the screening visit. Key exclusion criteria included type 1 or secondary diabetes, significant progression of diabetic macro- (acute cerebro-vascular event or any revascularization procedure, clinically-relevant peripheral artery disease, onset of a diabetic foot) or micro- (retinopathy progression, increase of at least 0.5 mg/dl of plasma creatinine or progression to macro-proteinuria, onset of clinically-relevant neuropathy) angiopathy in the 6 months prior to study visit. Other exclusion criteria included history of acute or chronic pancreatitis, pancreatectomy, gastric surgery, inflammatory bowel disease, organ failure or other severe diseases limiting life expectancy; drugs interfering with glucose levels (i.e. corticosteroids) or acute diseases (i.e. infections) in the 3 months before screening visit, history of inflammatory/infective/autoimmune chronic disease, contraindications to the maintenance of the background therapy (metformin), including—but not limited to—chronic kidney failure or plasma creatinine concentrations >1.5 mg/dl, severe respiratory failure, etc.; contraindications to the use of a sulfonylurea or DPP-4 inhibitors, clinically-relevant psychiatric disorders, any clinically significant abnormality identified in physical examination, laboratory tests (known chronic liver diseases, including HBV and HCV infection; liver markers above 2 times the upper normal limit) or vital signs at screening, and pregnancy.

### Study design

Eligible subjects were randomized in a 2:1 ratio to receive either V 100 mg/daily (50 mg bid) or G for 12 months. Treatment allocation and titration regimens were not blinded. Randomization was based on random numbers generated by a statistical software. Randomization numbers and sequence were kept by the data manager. Randomization list was concealed to study investigators. Principal investigator and collaborators investigator enrolled the participants. Drug treatment with G was started with 2.5 mg bid (daily total = 5 mg) 30 min before breakfast and dinner. After 1 month, G dose was up-titrated to a maximum of 7.5 mg/day and at month 4 up to a maximum of 5 mg bid to achieve pre-prandial finger-stick glucose values between 80 and 140 mg/dl (4.4–7.8 mmol/l) and post-prandial ≤200 mg/dl (11 mmol/l). No dose titration was planned for V. At baseline, medical history, current therapies, personal history of diabetes and CV disease, smoking and drinking habits were recorded. During the treatment period, subjects returned to the clinic for efficacy and safety assessments at 4 and 12 months. At baseline and at each following visit clinical parameters (BMI and systolic and diastolic blood pressure) and blood test [fasting plasma glucose, (FPG) HBA1C, plasma C-peptide, total cholesterol, triglycerides, HDL-cholesterol, liver enzymes, serum creatinine and uric acid] were performed. Estimated eGFR was calculated with the CKD-EPI formula [17]. In addition, circulating pro-inflammatory chemokine/cytokine profile [C-reactive protein (CRP), IL-6, TNF-∝], brain natriuretic peptide (BNP), total GLP-1, (C-term) SDF-1∝ and EPC number were also assessed. The study was conducted according to the guidelines of good clinical practice and the Declaration of Helsinki. The protocol and amendments were approved by the Parma Ethics Committee. All subjects provided written informed consent prior to study entry.

### Quantification of circulating endothelial progenitor cells

**Fig. 1:**
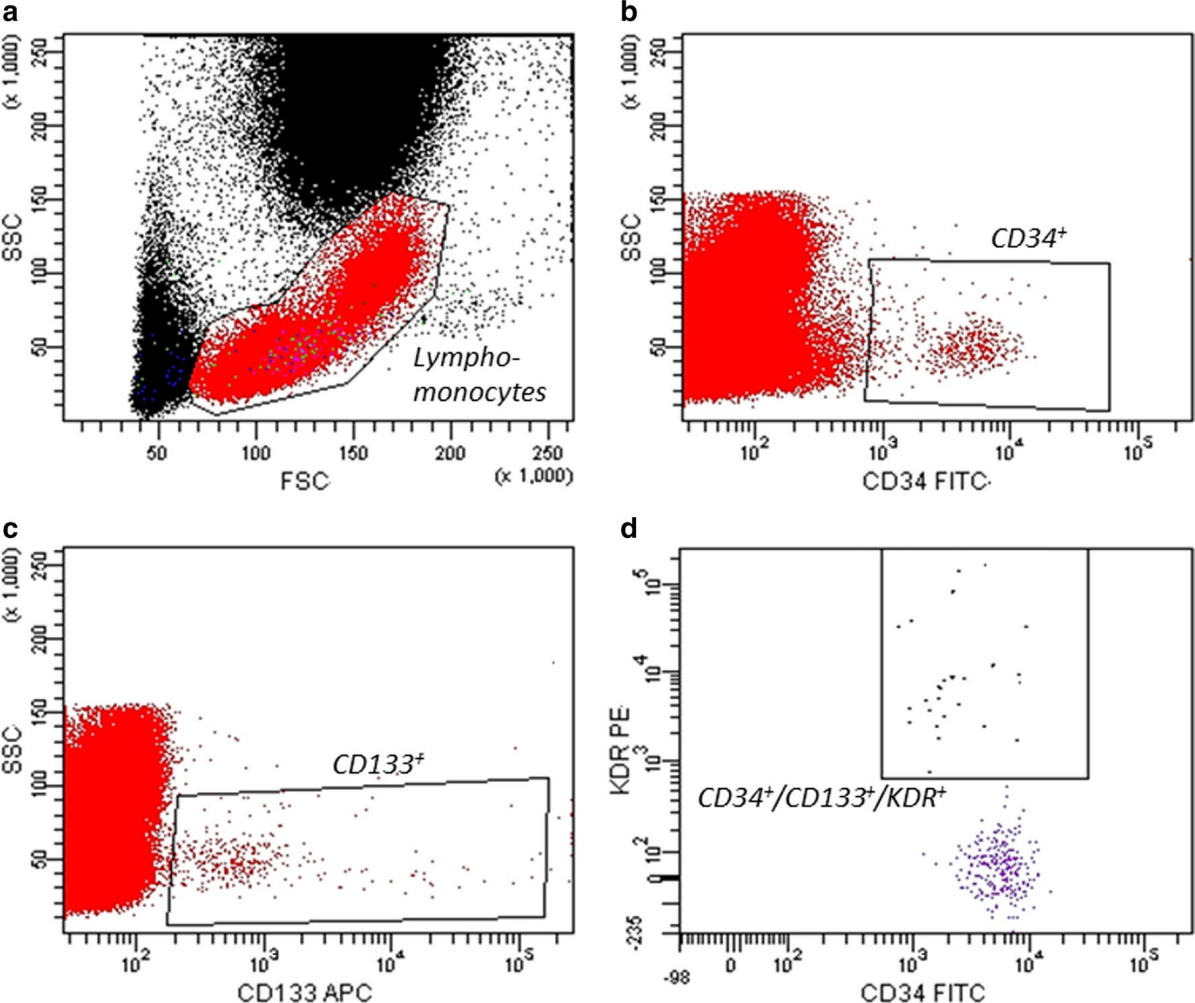
EPC number evaluation. Flow-cytometry analysis scatter plots to assess EPC number. Within the lymphomonocyte population (**a**) CD34 (**b**) and CD133 (**c**) positive cells were gated and evaluated for the expression of KDR to identify CD34^+^/CD133^+^/KDR^+^ cells (**d**)

Circulating EPCs were assessed by cytofluorimetric analysis for the expression of CD34, CD133, and kinase insert domain receptor (KDR) surface antigens: as previously described in detail [18–20]. Briefly, fasting blood samples were collected in EDTA and stained within 2 h with 10 μl fluorescein isothiocyanate (FITC)-conjugated anti-human CD34 monoclonal antibody (mAb) (Becton–Dickinson Biosciences, Franklin Lakes, NJ, USA), 20 μl allophycocyanin (APC)-conjugated anti-human CD133 mAb (Miltenyi Biotec, CA, San Diego, U.S.) 20 μl phycoerythrin (PE)-conjugated anti-human KDR mAb (R&D Systems, Minneapolis, MN, USA) and 20 μl peridinin chlorophyll protein complex (PerCP)-conjugated CXCR-4 (R&D Systems). Immunoglobulins were used as isotype controls (BD Biosciences). CD34^+^/CD133^+^/KDR^+^ cells were identified by evaluating the expression of KDR within the CD133^+^/CD34^+^ cells in the lymphomonocyte population (Fig. 1). The percentage of CD34^+^/CD133^+^/KDR^+^ cells co-expressing CXCR-4 was also assessed. Circulating EPCs (expressed as number of CD133^+^/CD34^+^/KDR^+^ cells/10^6^ total cytofluorimetric events) were acquired in a FACSCantoII cytometer and analysed using FACSDiva software (both by BD Biosciences). A single trained operator performed all cytofluorimetric analyses and was blinded to the patients’ status and allocation.

### (*C*-*term*) SDF-1α, IL-6, TNF α, CRP, BNP and total GLP-1 assessment

ELISA kits were used to measure plasma levels of (C-term) SDF-1α (Human CXCL12/SDF-1 alpha Quantikine ELISA Kit by R&D system), IL-6 (Human IL-6 ELISA Pro Kit by Mabtech, Nacka Strand, Sweden) and TNF-α (TNF alpha Ultrasensitive ELISA Kit, Thermo Fisher Scientific, Waltham, MA U.S.A.) following manufacturer’s instruction. Intra- and inter-assay coefficients of variation were 3.5 and 10.6% for SDF-1α, 2.5 and 3.8% for IL-6 and 6.0 and 8.5% for TNF-α, respectively. Cytokine/chemokine concentrations were calculated in duplicate by using a standard curve generated by serially diluting reconstituted standards and by measuring the absorbance at 450 nm in a microplate reader (Multiskan™ FC Microplate Photometer, Thermo Scientific).

Serum levels of extended range C-reactive protein were determined using the particle-enhanced turbidimetric immunoassay (PETIA technique; Siemens Dimension, USA). The reference range in this method is 0.0–5.0 mg/l. BNP levels were assessed using an Alere Triage reagent pack (Alere Inc., Ottawa, ON, CAN) and analysed in an automated D × I 800 immunoanalyzer (Beckman-Coulter, Fullerton, CA, USA).

Total GLP-1 was measured by a magnetic bead kit (Merck-Millipore, Vimodrone, Italy) according to kit instructions and analyzed on a MagPix (Luminex Corporation). The assay is based on the attachment of GLP-1 to magnetic beads and processing using LED excitation.

### Study endpoints

The study primary endpoint was the change from baseline values of the EPC number in the V vs G arm at 4 and 12 months. The secondary endpoint was the change from baseline values of the (C-term) SDF-1∝ levels in the V vs G arm at 4 and 12 months. Safety assessments included adverse events (AEs), incidence and severity of hypoglycemia [21], discontinuation for hyperglycemia (defined as HBA1C ≥ 8%; ≥63.9 mmol/mol) at 4 month, use of rescue medication for hyperglycemia, abnormal findings in physical exam and laboratory workup.

### Sample size determination

Sample size was calculated to achieve 80% power to reject the null hypothesis of equal mean changes in the primary endpoint when the population mean difference is 0.15 with a standard deviation of 0.2 in both groups and with a significance level (∝) of 0.05 using a two-sided two-sample equal-variance *t* test (difference between the two treatments at 12 months) (software PASS- NCSS, USA). In addition, 40 subjects were sufficient to guarantee a delta value between 0 and 12 months in the treatment group of ~10% (SD of pair differences 20%) with a ∝ value of 5% and β = 80% [22].

### Statistical analysis

Continuous variables are expressed as mean ± standard deviation or median (interquartile range IQR) for skewed distributed data. Categorical variables are expressed as frequencies. EPC values were log-transformed in order to achieve normal distribution as confirmed by Kolmogorov–Smirnov test. Comparisons of parameters at baseline between treatment groups were performed using the t test for normally distributed data, the Mann–Whitney test for non-normally distributed variables otherwise and the Chi square test for categorical variables.

Intra-groups differences within variables before and after treatment during the study have been analysed using a general linear model (GLM) for repeated measures with treatment (V vs G) and time (baseline and visits at 4 and 12 months) as main factors. Primary (EPC) and secondary (SDF-1α) endpoint analyses were performed by means of GLM with adjustment for baseline levels. Analysis was conducted according to the intention to treat (ITT) approach. A p value ≤0.05 was considered significant. Statistical analysis was performed using SPSS v. 22 (IBM Statistics).

## Results

### Study subjects

**Fig. 2:**
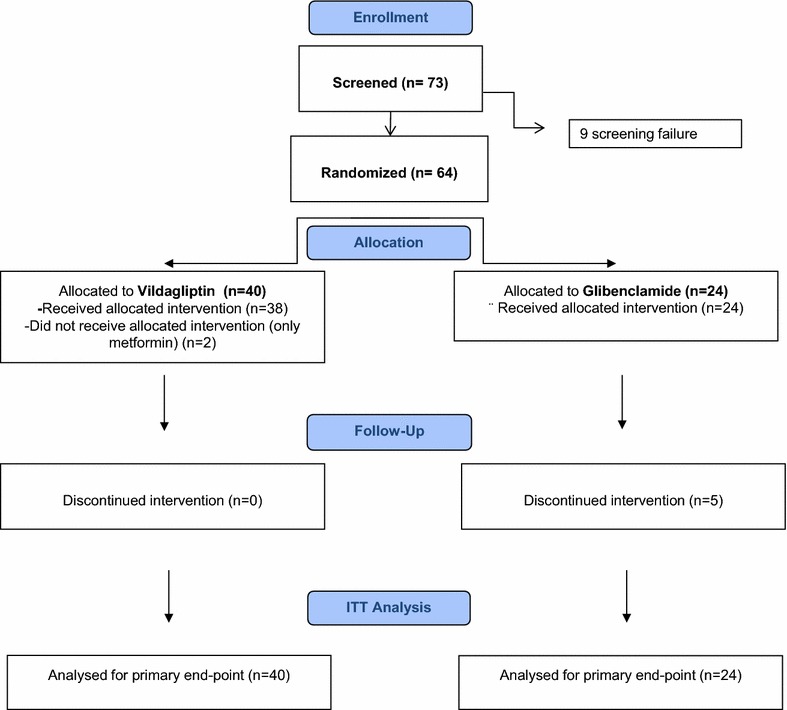
Consort flow chart. The *figure* shows the consort flow chart according to ITT analysis

Seventy-three individuals with type 2 diabetes entered the screening phase. Patient were screened and enrolled between September 2010 and December 2013 (last patient follow-up at 12 months in December 2014). Of these, 64 were randomised: 40 in the V arm and 24 in the G arm according to 2:1 ratio randomisation. Two subjects randomised to the V arm did not start the trial drug due to lack of compliance. None of the other subjects in the V arm discontinued the intervention in the 12 month follow-up, whereas in five subjects in the G arm the comparator drug was discontinued on the basis of the clinical judgment of their physician, owing to hypoglycemic (4 mild and 1 severe) events. The median duration of therapy was 12 (IQR 11–13) months without significant difference between study arms [12 (11–12) for V, 12 (7–13) for G]. Figure 2 shows the CONSORT flow chart according to ITT analysis . As expected by the randomisation procedure, baseline characteristics, concomitant therapies and metformin doses (mg/day) were similar in the two groups as shown in Table 1.

**Table 1:** Subject characteristics

Variables	Missing	Total (N = 64)	Vildagliptin (N = 40)	Glibenclamide (N = 24)	p
Age (years)	0	62 ± 9	61 ± 9	63 ± 10	0.28
Gender male n (%)	0	43 (67)	26 (65)	17 (71)	0.63
Smoking habit n (%)	3	13 (22)	7 (19)	6 (26)	0.72
CVD history n (%)	2	13 (20)	8 (20)	5 (21)	0.94
Hypertension n (%)	2	43 (67)	25 (63)	18 (75)	0.40
Disease duration (yrs)	0	6.5 (3–10)	7 (4–11)	5 (1–10)	0.30
Plasma glucose (mg/dl)	0	154 ± 34	155 ± 36	150 ± 30	0.67
HBA1C (%)	0	7.7 (7.4–8.1)	7.7 (7.4–7.9)	7.7 (7.5–8.1)	0.57
HBA1C (mmol/mol)	0	61 (57–65)	61 (57–64)	61 (58–65)	0.57
Plasma C-peptide (ng/ml)	8	2.67 (2.16–3.52)	2.66 (2.07–3.48)	2.78 (2.21–4.34)	0.69
BMI (kg/m^2^)	0	29.0 (26.2–33.9)	29.1 (26.8–32.9)	28.9 (25.4–34.1)	0.94
Serum creatinine (mg/dl)	2	0.8 (0.6–0.9)	0.8 (0.6–0.9)	0.8 (0.6–1.0)	1.0
GFR (CKD-EPI) (ml/min/1.73 m^2^)	3	96.0 ± 12.7	96.1 ± 11.8	96.0 ± 14.5	0.77
Total cholesterol (mg/dl)	2	166.6 ± 30.3	168.5 ± 31.1	163.2 ± 29.8	0.56
HDL-cholesterol (mg/dl)	2	45 (40–54)	45 (41–51)	45 (37–55)	0.82
Tryglicerides (mg/ml)	2	111 (71–155)	100 (68–140)	117 (78–1182)	0.48
GGT (U/l)	4	20 (16–32)	19 (16–30)	23 (14–36)	0.63
AST (U/l)	4	23 (19–28)	23 (18–27)	23 (19–28)	0.89
ALT (U/l)	4	27 (19–38)	27 (19–38)	28 (20–39)	0.63
Uric acid (mg/dl)	4	5.2 (4.5–6.0)	5.2 (4.5–5.9)	5.2 (4.4–6.3)	0.76
EPC (n/10^6^ events)	0	38.0 (24.2–58.2)	39.0 (24.0–58.2)	37.5 (25.0–59.8)	0.81
Therapies					
Anti-hypertensive n (%)	2	43 (67)	25 (64)	18 (75)	0.40
Lipid-lowering n (%)	2	44 (69)	26 (67)	18 (75)	0.33
Antiplatelet n (%)	2	30 (47)	20 (51)	10 (42)	0.40
Metformin (mg/day)	0	2000 (1750–2735)	2000 (2000–2550)	2000 (1700–2400)	0.13

Baseline characteristics of the study subjects expressed as (mean ± SD) or median (IQR) for continuous data and n (%) for categorical data

### Effect of treatment on clinical parameters

As expected, both therapies significantly (GLM p < 0.0001) and similarly (p = 0.92) improved glucose control during the 12-months follow-up (Table 2). Accordingly, FPG was significantly and similarly reduced during the follow-up in both arms (p < 0.0001). Thus, glucose equipoise between the two study arms was achieved.

**Table 2:** Results of GLM model

	Vildagliptin	Glibenclamide	Treatment effect	Time effect	Interaction
HBA1C (%)					
Baseline	7.7 (7.4–7.9)	7.7 (7.5–8.1)	p = 0.85	*p* < *0.001*	p = 0.17
4 months	6.8 (6.4–7.3)	6.8 (6.4–7.3)			
12 months	7.0 (6.5–7.3)	7.1 (6.5–7.5)			
FPG (mg/dl)					
Baseline	155 ± 36	150 ± 30	p = 0.80	*p* < *0.001*	p = 0.64
4 months	130 ± 36	129 ± 28			
12 months	129 ± 28	129 ± 30			
BMI (kg/m^2^)					
Baseline	29 (27–33)	29 (25–34)	p = 0.80	p = 0.67	p = 0.79
4 months	29 (26–33)	28 (26–33)			
12 months	24 (26–33)	29 (26–34)			
eGFR (ml/min/1.73 m^2^)					
Baseline	96.1 ± 11.8	96.0 ± 14.5	p = 0.24	*p* = *0.039*	p = 0.55
4 months	95.8 ± 10.5	92.3 ± 14.7			
12 months	95.8 ± 12.0	92.5 ± 12.9			
EPC (n/10^6^ events)					
Baseline	39.0 (24.0–58.2)	37.5 (25–59.8)	*p* = *0.025*	*p* = *0.008*	p = 0.58
4 months	37.0 (27.0–65.0)	36.0 (23.0–54.2)			
12 months	45.0 (29.7–67)	32.0 (22.2–53.5)			
Circulating markers					
SDF-1α (pg/ml)					
Baseline	2877 (2388–3713)	3134 (2763–4117)	*p* < *0.001*	p = 0.38	*p* = *0.035*
4 months	2293 (1585–3187)	3100 (2572–3782)			
12 months	2064 (1540–2954)	3336 (2778–3863)			
BNP (pg/ml)					
Baseline	3 (1–9)	3 (2–7)	p = 0.41	*p* = *0.045*	p = 0.71
4 months	3 (1–8)	3 (1–5)			
12 months	5 (3–12)	6 (3–10)			
IL-6 (pg/ml)					
Baseline	4.7 (3.0–7.6)	6.1 (4.2–9.2)	p = 0.93	p = 0.77	p = 0.15
4 months	5.8 (3.7–9.7)	5.4 (3.5–8.2)			
12 months	5.0 (2.7–9.9)	5.4 (3.7–6.7)			
CRP (mg/l)					
Baseline	4.0 (3.2–5.7)	3.8 (2.6–6.3)	p = 0.43	p = 0.51	p = 0.79
4 months	4.0 (2.7–5.5)	3.2 (2.4–6.3)			
12 months	3.6 (2.3–6.1)	3.5 (2.9–3.9)			
TNFα (pg/ml)					
Baseline	1.4 (0.7–2.0)	1.6 (1.1–2.4)	p = 0.24	p = 0.25	p = 0.97
4 months	1.1 (0.5–1.8)	1.7 (1.3–2.4)			
12 months	1.1 (0.4–1.8)	1.4 (0.8–2.2)			
GLP-1 total (pM)					
Baseline	84.0 (66.2–109.0)	86.7 (73.7–97.0)	p = 0.75	p = 0.83	p = 0.91
4 months					
12 months	83.4 (68.8–107.0)	88.5 (75.5–114.6)			

Data are reported as mean ± standard deviation or median (IQR)

Clinical variables, circulating markers and p values of GLM models (treatment, time and time × treatment effects)

GLM model for primary (EPC) and secondary (SDF-1α) endpoint was performed with adjustment for baseline levels

Statistically significant values (p < 0.05) are shown in italics

A small, albeit statistically significant, decrease in eGFR (p < 0.05) occurred during the follow-up in both study arms. No significant differences in other study parameters (i.e. BMI and lipid profile) with respect to treatment and time were observed (Table 2).

### Effect of treatment on primary and secondary endpoints

**Fig. 3:**
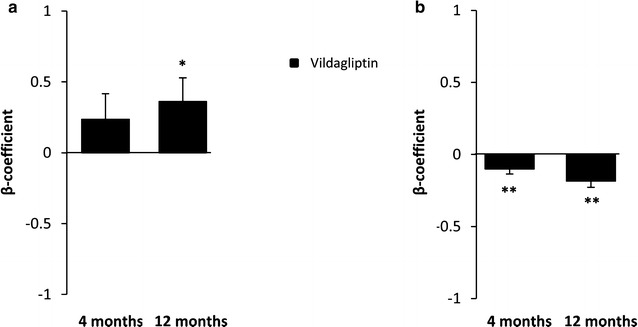
Treatment effect. The figure displays the treatment effect (β-coefficient and SE) of vildagliptin (treating glibenclamide as reference group) on EPC number (**a**) and SDF-1α levels (**b**) in repeated measure GLM models after adjustment for baseline values. (*p < 0.05; **p < 0.01)

EPCs and (C-term) SDF-1α levels in the two arms during the study and both time and treatment effects -and their interaction- according to the GLM model are shown in Table 2. Vildagliptin treatment significantly increased EPC number at 12 months (p = 0.034; β = 0.362; SE 0.166) compared to glibenclamide after adjusting for baseline EPC values as shown in Fig. 3a (GLM model). No significant between-arm difference in EPC count was found at 4 months (p = 0.196; β = 0.236; SE 0.180). At baseline EPC were 91.2% (V) and 95.9% (G) positive for CXCR-4 expression, with no between-arm significant differences at 4 and 12 months. Vildagliptin treatment significantly reduced (C-term) SDF-1α plasma levels at 4 (p = 0.004; β = −0.102; SE = 0.034) and 12 months (p < 0.001; β = −0.185; SE = 0.043) compared to the G arm after adjustment for baseline values (GLM) (Fig. 3b).

### Other assays

IL-6, TNF-α and CRP plasma levels were stable throughout the study in both groups. BNP slightly and equally increased in both study arms (p < 0.05) (Table 2). No differences in total GLP-1 plasma levels were observed before and after treatment in both study arms (Table 2).

### Adverse events

Five out of 24 (21%) patients in the G arm experienced hypoglycaemia: mild hypoglycemic events (n = 4) and severe hypoglycemic (n = 1) event occurred at month 1 (0.5–2.5) after randomisation. These events led to treatment discontinuation. No adverse events were reported in the V arm during the whole study period.

## Discussion

Our findings show that vildagliptin, but not glibenclamide, exerts a beneficial long-term (12-month) effect on circulating EPC levels at glucose equipoise in individuals with type 2 diabetes. Since a reduced EPC number is a biomarker and, perhaps, a pathogenetic factor of vascular disease, DPP-4 inhibition by vildagliptin may represent an attractive strategy to treat diabetes, beyond glucose control.

The effects of DPP-4i on EPC bioavailability were reported in short-term studies. The first evidence indicated that a 4-week treatment with sitagliptin increased circulating EPC number (CD34^+^ and KDR^+^ cells) in a non-randomised study conducted in a small group of patients with type 2 diabetes [9]. The same authors reported the acute effects (4-days) of linagliptin administration in increasing circulating EPC number vs placebo in a group of individuals with type 2 diabetes, of whom 39% had concomitant chronic kidney disease (CKD) [10].

In a very recent paper, a mid-term (4-month) treatment with sitagliptin was associated with a significant increase in EPCs -phenotypically characterised as CD34^+^/CXCR-4^+^ cells- in 30 patients with type 2 diabetes in poor glucose control with metformin and/or sulphonylurea [11]. However, close scrutiny of the reported data raises the possibility that statistical significance might be also attributable to the effect of regression to the mean. In addition, in that study the improvement in glucose control was greater with sitagliptin compared to the comparator (glimepiride), thereby potentially implying that the effects of sitagliptin may be mediated by improved glucose control.

Indeed, glucose lowering per se can increase EPC bioavailability [23, 24]. Thus, the long-term effect of DPP-4 inhibition on systemic EPC bioavailability, independently of glucose control, remained somewhat uncertain.

Our study expands the hitherto findings in a larger randomised-controlled long-term trial, with a DPP4-i—vildagliptin-, thus far unexplored as to these biologic effects. The results document a positive direct effect of vildagliptin treatment on EPC bioavailability at glucose equipoise, definitely ruling out glucose control as a confounder. The effect of DPP-4 inhibition in raising EPC levels was sustained for 12 months, strongly suggesting a long term beneficial effect of this therapy on the endothelial repair process and its counterbalancing role to endothelial injury.

These findings might partially explain the DPP-4i-mediated beneficial vascular effects which have been reported in pre-clinical [25–27] and clinical studies [28, 29].

However, it should be noticed that large intervention trials have failed to demonstrate any additional protective CV effect of this class of drugs compared to active comparators at glucose equipoise [30–32]. These trials have been conducted in high risk populations with diabetes, (∼80% with a history of CV disease in the saxagliptin and cardiovascular outcomes in patients with type 2 diabetes mellitus -SAVOR-TIMI-, with recent acute coronary syndrome in the alogliptin after acute coronary syndrome in patients with type 2 diabetes -EXAMINE- and with established CV disease in the effect of sitagliptin on cardiovascular outcomes in type 2 diabetes -TECOS- studies), with diverse median follow-up durations (from 19 months to 3 years). Long-term potential CV benefits in patients with type 2 diabetes at lower risk have not been investigated. It is possible, albeit not proved, that in patients in a less advanced stage of the natural history of CV damage, such as those included in the present study, DPP-4 inhibition can provide protection against CV events. Noteworthy, the beneficial vascular effects of DPP-4i may be mirrored first by a microvascular improvement, subsequently leading to a long-term macrovascular benefit [33, 34]. Accordingly, EPC levels predict microvascular outcomes in type 2 diabetes [35], suggesting that the DPP-4i-induced reduction in microalbuminuria progression [36] might be also mediated by an increase in EPC number; this hypothesis, although plausible, needs to be proven.

The chemokine SDF-1α is a substrate of DPP-4. Hence, DPP-4 inhibition might increase SDF-1α bioavailability, by restraining DPP-4-mediated degradation. However, SDF-1α is also emerging as a novel player among CV risk indicators/factors. SDF-1α is a pivotal mediator of stem cell mobilisation from the bone-marrow niches and of their homing to ischemic sites, promoting angiogenesis and vasculogenesis. This beneficial action is particularly relevant in experimental conditions of ischemia [37] or of injury-induced restenosis [38]. In humans, the results are controversial [39, 40]. In contrast, the role played by SDF-1α in atherogenesis/atherosclerosis might be different. SDF-1α is released by the atherosclerotic plaques, it binds to its receptor which is expressed in inflammatory cells (i.e. monocytes and macrophages), and it can promote chemotaxis, leukocyte recruitment and platelet aggregation [41], which result into accelerated plaque formation [42]. Furthermore, circulating (C-term) SDF-1α has emerged as an independent cardiovascular risk biomarker in large (>3000 individuals) prospective studies. In the Framingham Heart study, which included 10% of individuals with diabetes, higher SDF-1α values were directly and independently associated with the risk of heart failure and 10-year all-cause mortality [14] and inversely with CD34^+^ (bone marrow-derived) circulating cell phenotypes. Consistently, higher SDF-1α levels independently predicted incident myocardial infarction and death in a large cohort of high risk subjects with CKD (50% having diabetes) at 6-year follow-up [15].

Thus, the vildagliptin-induced reduction in plasma SDF-1α levels observed in this study should be interpreted in the context of the assessment of CV risk and not as a possible explanation of the DPP-4i mediated increase in EPC number.

Studies assessing SDF-1α in the course of treatment with DPP-4i have reported either an increase or a decrease in the chemokine plasma levels [9–11]. These apparently discrepant findings of DPP-4 inhibition on circulating SDF1α levels can be due to methodological differences in SDF-1 assays. The assays used in our study, and in the epidemiological studies reporting a direct relationship between SDF-1α levels and CV risk, as is the above mentioned epidemiological studies [14, 15], are selective for the C-terminus of SDF-1α and measure total SDF-1α (active and non-active). In contrast, in those studies in which DPP-4 inhibition was reported to increase SDF-1 levels a custom assay specific for the N-terminus of SDF-1, which contains the active receptor (CXCR-4)-binding site and the X-Pro dipeptide cleaved by DPP-4, was used and only active SDF-1 was measured [9, 10]. While a role of the increase in the SDF-1α bioactive form brought about by DPP-4 inhibition still needs to be firmly established, robust epidemiological evidence demonstrates that the total (C-term) form is independently associated to incident CV disease and mortality. In analogy with the DPP-4/GLP-1 axis, we cannot exclude that a decrease in the total (C-term) SDF-1 with DDP-4i may be paralleled by an increase in the bioactive form and vice versa [43].

Two additional findings of our study deserve to be commented.

BNP increased and eGFR decreased with no difference between study arms. It should be noted that in both cases the changes were so small as to be of questionable clinical relevance, and could not be ascribed to vildagliptin treatment.

The putative mechanisms underlying DPP-4i ability to increase EPC bioavailability are beyond the scope of our study. However, the decrease in (C-term) SDF-1α plasma levels with DPP-4 inhibition, while it does not necessarily negate a SDF-1α mediated mechanism, is not consistent with it. DPP-4 inhibition is endowed with robust anti-inflammatory actions in cellular and mouse models which may explain the majority, if not all, of the pleiotropic effect of this class of drugs [44], possibly including the amelioration in EPC biology. However, data on DPP-4i effect on plasma cytokine levels are scarce in humans [45] and the unchanged cytokine and CRP levels in our study seem not to support this hypothesis.

Strengths of our study include the controlled-randomised study design, the relative large population involved, which has been extensively characterised, and, importantly, the 1-year follow-up, which ensures the demonstration of a durable DPP-4i effects on boosting EPC bioavailability. In addition, our is the first evidence of a beneficial effect on EPC caused by vildagliptin treatment.

Some limitations of our study should be recognized. Although we demonstrated that vildagliptin improves EPC bioavailability, the underlying pathophysiological explanation remains unclear and future studies are warranted to unravel SDF-1α dependent and independent mechanisms. The clinical impact of the EPC and SDF-1α changes induced by vildagliptin, although concordantly pointing to a potential beneficial effect, remains unknown. The open-label nature of the study also needs to be acknowledged.

In conclusion, vildagliptin exerts a beneficial long-term effect on circulating EPC levels, at glucose equipoise, with a putative positive effect on vascular integrity. The vildagliptin-induced reduction in plasma SDF-1α levels might be desirable in light of the emerging role of circulating SDF-1α as an independent cardiovascular risk biomarker.

## Abbreviations

APC: allophycocyanin
BNP: brain natriuretic peptide
CKD: chronic kidney disease
CRP: C-reactive protein
CV: cardiovascular
DPP-4i: dipeptidyl peptidase-4 inhibitors
EPC: endothelial progenitor cells
FITC: fluorescein isothiocyanate
FPG: fasting plasma glucose
GLM: general linear model
GLP-1: glucagon-like peptide-1
ITT: intention to treat
KDR: kinase insert domain receptor
mAb: monoclonal antibody
PE: phycoerythrin
PerCP: peridinin chlorophyll protein complex
